# SubMito-PSPCP: Predicting Protein Submitochondrial Locations by Hybridizing Positional Specific Physicochemical Properties with Pseudoamino Acid Compositions

**DOI:** 10.1155/2013/263829

**Published:** 2013-08-21

**Authors:** Pufeng Du, Yuan Yu

**Affiliations:** ^1^School of Computer Science and Technology, Tianjin University, Tianjin 300072, China; ^2^Tianjin Key Laboratory of Cognitive Computing and Application, Tianjin University, Tianjin 300072, China

## Abstract

Knowing the submitochondrial location of a mitochondrial protein is an important step in understanding its function. We developed a new method for predicting protein submitochondrial locations by introducing a new concept: positional specific physicochemical properties. With the framework of general form pseudoamino acid compositions, our method used only about 100 features to represent protein sequences, which is much simpler than the existing methods. On the dataset of SubMito, our method achieved over 93% overall accuracy, with 98.60% for inner membrane, 93.90% for matrix, and 70.70% for outer membrane, which are comparable to all state-of-the-art methods. As our method can be used as a general method to upgrade all pseudoamino-acid-composition-based methods, it should be very useful in future studies. We implement our method as an online service: SubMito-PSPCP.

## 1. Introduction

Mitochondrion is a type of membrane-enclosed subcellular organelle that can be found in most eukaryotic cells [1]. It is involved in many biological processes, such as energy metabolism, programmed cell death, and ionic homeostasis [2]. Every mitochondrion can be divided into four subcompartments, including inner membrane, outer membrane, intermembrane space, and the matrix. The proteins in mitochondria can vary in different tissues and organisms. For example, human mitochondria may contain about 600 different proteins [3], while over 900 proteins were found in mouse mitochondria [4]. Mitochondria have been reported to be related in several human diseases and may play an important role in the aging process [5].

Computational identification of protein subcellular locations has become a challenge in the last decade. Recently, the research in this area focused on four different topics: (1) the prediction of multisites protein subcellular localization [6–9]; (2) the prediction of protein sub-subcellular locations [10], including the prediction of protein subnuclear locations, submitochondrial locations, and subchloroplast locations; (3) the prediction of topology-specific protein subcellular locations [11, 12]; and (4) the prediction of conditional mislocated protein subcellular locations [13]. Several promising results have been achieved in these four topics. Li et al. did a serial of interesting work to predict multisites protein subcellular localization by introducing the multilabel classification methods [14–16]. Lin et al. presented a serial of impressive results in predicting protein submitochondrial and subchloroplast locations [17, 18]. They also achieved great success in applying computational approaches in identifying Golgi-resident protein types as well as mycobacterial membrane protein types [19, 20].

Over the last few years, several studies focused on reporting computational methods to predict protein submitochondrial locations. Du and Li started this topic by proposing the SubMito system and the first benchmarking dataset [21]. Nanni and Lumini introduced a genetic-algorithm-based method to select sequence-based protein descriptors [22]. Shi et al. introduced the wavelet-SVM method to improve the prediction performance [23]. Fan and Li proposed a hybrid method using six different types of descriptors with incremental diversity algorithm as a feature selection procedure [24]. Zakeri et al. employed anther hybrid method to incorporate sequence-based descriptors, functional domain descriptors, and secondary structure information [25]. Lin et al. proposed to use the overrepresented tetrapeptides to predict the protein submitochondrial locations [17]. All of these methods improved the prediction accuracy on the same benchmarking dataset [26, 27].

With the increment of prediction accuracy, the complexity of algorithms and the dimensions of the feature vector to represent the protein sequence are also increasing. Du and Li started this topic by using 1080 dimensional vectors. Nanni and Lumini created 15 artificial features by combining several hundreds of different features. Shi et al. employed the discrete wavelet transformation and summary statistics to reduce the dimensions of features. Fan and Li introduced thousands of original features and used incremental diversity algorithm to reduce them to 613 dimensions. Zakeri et al. combined over a thousand different features in their method. Lin et al. used 160,000 original features and reduced them to 1302 dimensions using a confidence parameter. Except, SubMito, all the state-of-the-art methods were using different machine-learning-based algorithms to reduce the feature dimensions. It seems that the key to improve the prediction performance is to choose the right dimension reduction algorithms. 

Although the dimension reduction algorithms are consolidated based on statistics and are supported well by the underlying mathematical theories, it is usually difficult to reason the selected dimensions in a biological sense. We admit that the dimension reduction algorithms are effective and useful. It should be regarded as a powerful tool to improve the prediction performance of bioinformatics predictors. However, in this paper, we would present a method that can produce comparable prediction performance with only about 100 dimensions of features and without using any dimensional reduction algorithm.

## 2. Materials and Methods

### 2.1. Datasets

There are several datasets existing for predicting submitochondrial locations. These datasets are always extracted from UniProt database with several filtering procedures. Since the methods, which were proposed along with these datasets, may have different requirement to the dataset, there are differences in the filtering procedures. In order to reflect the most recent advances in the available data as well as demonstrating the prediction power of the current method, two datasets were adopted in the current study. One dataset was directly extracted from the most recent version of UniProt database, and the other is the SubMito dataset that was published by Du and Li.

The procedures for filtering the raw data from UniProt database are described as follows: First, the reviewed sequences in the UniProt database, which are annotated with subcellular location “mitochondrion,” were retrieved using the UniProt online query and retrieval system. Secondly, the sequences were screened to ensure every sequence has a uniquely annotated submitochondrial location among the four locations: mitochondrial inner membrane, mitochondrial outer membrane, mitochondrial matrix, and mitochondrial intermembrane space. Due to the limited number of multi-sites submitochondrial proteins, we do not consider them in the current study. Thirdly, the sequences which are fragment of other proteins are excluded. The remaining sequences are processed using the CD-HIT program to remove the highly homologues sequences. The identity cutoff was set to 40% in the CD-HIT program. Finally, the submitochondrial locations, which contain less than 15 sequences, were discarded. The remaining 983 sequences compose the dataset of this study. Among the 983 sequences, there are 661 sequences from inner membrane, 177 sequences from matrix, and 145 sequences from outer membrane. We use this dataset as the basis to train and test our method. This dataset was denoted as the SML3-983 dataset in the current study. 

The dataset of SubMito was also adopted as the basis for comparing the performance of our method to other existing methods, as all existing methods reported jackknife test performance on this dataset. The SubMito dataset contains 317 protein sequences from 3 submitochondrial locations, including 131 sequences from inner membrane, 41 sequences from outer membrane and 145 sequences from matrix. The pairwise sequence similarity in the dataset is lower than 40%. This dataset was denoted by the SML3-317 dataset in the current study. The summary of both datasets is shown in Table 1.

### 2.2. Sequence Representations

In order to improve the performance in predicting protein subcellular localizations, one of the keys is to represent the protein sequences with an effective discrete numerical form, which is able to reflect the intrinsic correlation with their localizations [28]. The PseAACs (pseudoamino acid compositions) have been commonly used to represent protein sequences in predicting their subcellular locations [29]. It is also extended recently to represent nucleotide sequences as well [30]. The basic idea of the PseAAC is to extract the sequence order information with the autocorrelation coefficients of the protein sequence if every residue on the protein sequence can be represented with a number [31]. The physicochemical properties of amino acids, like hydrophobicity and hydrophilicity values, were used for this purpose [32]. 

Biology is a natural science with historical dimensions. In the evolution history, the mutations in DNA level may produce the changes of single residues or insertion or deletion of several residues on the protein sequences. However, the function and the localization of the protein may remain unchanged. Therefore, we should investigate a group of evolutionary related protein sequences rather than a single protein sequence, which will make it easy to determine which residues are relatively more important in preserving the function and the localization of the protein. In recent years, the PsePSSM (pseudopositional specific scoring matrix), which applies the pseudoamino acid composition concept on the PSSM (positional specific scoring matrix), was widely applied in representing protein sequences [33–36]. 

Next, we propose a method that replaces the physicochemical properties in the PseAAC with the PSPCP (positional specific physicochemical properties), which can be derived from the PSSM and the existing physicochemical properties. 

Let *P* = *R*_1_ *R*_2_ ⋯ *R*_*L*_ be a protein sequence with length *L*, where *R*_1_,*R*_2_,…,*R*_*L*_ are the *L* residues on the protein sequence. By searching *P* against the SwissProt database using PSI-BLAST program [37] with three iterations and 0.001 as the e-value threshold, a PSSM can be produced as follows:

(1) $$\begin{array}{c} E\left(P\right)=\left[\begin{array}{cccc} E_{1\to 1} & E_{1\to 2} & \cdots & E_{1\to 20}\\ E_{2\to 1} & E_{2\to 2} & \cdots & E_{2\to 20}\\ \vdots & \vdots & \ddots & \vdots \\ E_{L\to 1} & E_{L\to 1} & \cdots & E_{L\to 20} \end{array}\right], \end{array}$$

where *E*_*i*→*j*_ is a score generated by the PSI-BLAST. This score described the propensity of the *i*th residue on the protein sequence that is being changed to the *j*th type of amino acid during the evolutionary process. 

Because of the PSSM generation process in PSI-BLAST, this number can be either positive or negative. It can also vary in a large range. In order to make every element in (1) within the range [0,1], a conversion was performed to create a standardized matrix as follows:

(2) $$\begin{array}{c} A\left(P\right)=\left[\begin{array}{cccc} A_{1\to 1} & A_{1\to 2} & \cdots & A_{1\to 20}\\ A_{2\to 1} & A_{2\to 2} & \cdots & A_{2\to 20}\\ \vdots & \vdots & \ddots & \vdots \\ A_{L\to 1} & A_{L\to 1} & \cdots & A_{L\to 20} \end{array}\right], \end{array}$$

where

(3) $$\begin{array}{c} A_{i\to j}=\frac{\exp\left(E_{i\to j}\right)}{\sum_{j=1}^{20}\exp\left(E_{i\to j}\right)},\;i=1,2,\ldots ,L;\;\;j=1,2,\ldots ,20. \end{array}$$

Let *H*(*r*, *j*) be the *r*th physicochemical property of the *j*th type of residue. We now use the *r*th physicochemical property to derive a PSPCP for *R*_*i*_ on the protein sequence *P*, as given by

(4) $$\begin{array}{c} d_{i,r}\left(P\right)=\sum_{j=1}^{20}A_{i\to j}h\left(r,j\right), \end{array}$$

where *d*_*i*,*r*_(*P*) is the PSPCP derived from the *r*th physicochemical property for *R*_*i*_ and *h*(*r*, *j*) is the normalized *r*th physicochemical property of the *j*th type of residues. It can be computed as follows:

(5) $$\begin{array}{c} h\left(r,j\right)=\frac{H\left(r,j\right)-m\left(r\right)}{s\left(r\right)}, \end{array}$$

where

(6) $$\begin{array}{c} m\left(r\right)=\frac{1}{20}\sum_{j=1}^{20}H\left(r,j\right),\\ s\left(r\right)=\sqrt{\frac{1}{20}\sum_{j=1}^{20}{\left(H\left(r,j\right)-m\left(r\right)\right)}^{2}}. \end{array}$$

We now use the PSPCP to replace the physicochemical properties in the amphiphilic pseudoamino acid compositions (AmPseAAC) [31]. We compute the following twenty descriptors to replace the amino acid compositions in the AmPseAAC:

(7) $$\begin{array}{c} f_{j}\left(P\right)=\frac{1}{L}\sum_{i=1}^{L}A_{i\to j},\;j=1,2,\ldots ,20. \end{array}$$

The pseudofactor that describes the *k*th tier sequence-order effect with the PSPCP, which is derived from the *r*th physiochemical property, can be formulated as (8)

(8) $$\begin{array}{c} u_{k,r}\left(P\right)=\frac{1}{L-k}\sum_{i=1}^{L-k}d_{i,r}\left(P\right)d_{i+k,r}\left(P\right). \end{array}$$

Given the parameters, *w* and *λ*, and *R* types of physicochemical properties, we create 20 + *λR* descriptors for protein *P* as follows:

(9) qn(P)={fn(P)∑n=120fn(P)+w∑r=1R∑k=1λuk,r(P),      1≤n≤20wuk,r(P)∑n=120fn(P)+w∑r=1R∑k=1λuk,r(P),    n=20+(r−1)λ+k,    1≤k≤λ,  1≤r≤R,

where *w* should be in the range (0,1) and *λ* can be a positive integer less than the length of the shortest sequence in the benchmarking dataset. 

The protein *P* can be represented as a 20 + *λR* dimension vector as

(10) $$\begin{array}{c} Q\left(P\right)={\left[q_{1}\left(P\right),q_{2}\left(P\right),\ldots ,q_{20+\lambda R}\left(P\right)\right]}^{T}. \end{array}$$

When the PSSM is not available, *A*_*i*→*R*_*i*__ = 1 would be assumed. The whole sequence representation would automatically degrade to AmPseAAC.

### 2.3. Prediction Algorithm

We use SVM (support vector machine) as the prediction algorithm in this study. It searches for an optimal separating hyperplane, which maximizes the margin in feature space [38]. We used an RBF (radial basis function) kernel in this study, as the RBF kernel is the most flexible and the most widely used kernel function. The RBF kernel function can be formulated as follows:

(11) $$\begin{array}{c} K\left(Q\left(P_{x}\right),Q\left(P_{y}\right)\right)=\exp\left(-\gamma {\left|Q\left(P_{x}\right)-Q\left(P_{y}\right)\right|}^{2}\right), \end{array}$$

where *γ* is a parameter, **Q**(*P*_*x*_) and **Q**(*P*_*y*_) are 20 + *λR* dimension vectors representing proteins *P*_*x*_ and *P*_*y*_, and “|·|” is the operator that computes the Euclidean length of a vector.

### 2.4. Performance Evaluations

The jackknife test, which is deemed to be the most objective and rigorous protocol for evaluating predictive bioinformatics methods, was applied in evaluating the performance of our method [39]. The following summary statistics were used to measure the prediction performance:

(12) Accs=TPsTPs+FNs, s=1,2,3,MCCs=TPsTNs−FPsFNs(TPs+FPs)(TPs+FNs)(TNs+FPs)(TNs+FNs),s=1,2,3,ACC=∑s=13TPs∑s=13TPs+FNs,

where Acc_*s*_ is the prediction accuracy for the *s*th location, MCC_*s*_ is the Mathew's correlation coefficient [40] for the *s*th location, ACC is the overall prediction accuracy, and TP_*s*_, TN_*s*_, FP_*s*_, and FN_*s*_ are the numbers of true positives, true negatives, false positives, and false negatives of the *s*th location in the jackknife test, respectively.

### 2.5. Parameter Calibrations

There are several parameters in our method. The value of these parameters will affect the prediction performance of our method. These parameters were calibrated to optimize the jackknife test overall accuracy. Nine different types of physicochemical properties, which are the same as the SubMito method, were applied in this method. These physicochemical properties can be found in Table 2. The parameters *w* and *λ* were selected by enumerations. The parameter *w* was enumerated in the range 0.05 to 1.0 with step 0.05. The parameter *λ* was enumerated in the range 2 to 20 with step 1. Altogether, 380 combinations of *w* and *λ* were tested. For every combination, a grid search was carried out using LIBSVM software package [41] to optimize the jackknife test performance by finding the best values of the parameters *γ* and *C*, which are the cost parameters in training SVM models. 

## 3. Results and Discussions

### 3.1. Prediction Performance

The jackknife test on SML3-983 dataset was shown in Table 3. The optimal performance was achieved when *w* = 0.15, *λ* = 11, *γ* = 0.125, and *C* = 8. The optimal jackknife test performance on SML3-983 was 89.01%.

Since all existing methods reported their jackknife test performance on SML3-317 dataset, we also optimized our method on that dataset for a performance comparison. On SML3-317 dataset, we achieved the best performance when *w* = 0.15, *λ* = 9, *γ* = 0.125, and *C* = 2. The optimal performance of our method on SML3-317 was listed in Table 4 with the comparison to the other existing methods.

On SML3-317 dataset, the overall prediction accuracy of our method achieved over 93%, which is comparable to all state-of-the-art methods. Obviously, some other methods have achieved about 1% higher overall accuracy than our method. Nevertheless, no existing method achieved better prediction accuracy on all three submitochondrial locations. It should also be noticed that our method achieved 98% accuracy on the inner membrane class, which is higher than SubIdent, MitoLoc, and Fan and Li's method. The only method that has higher prediction accuracy on the inner membrane class is the TetraMito. However, TetraMito has a lower MCC value on the inner membrane class, which indicates that the 100% accuracy on the inner membrane class may be on the cost of decreasing accuracy of the other locations. As anticipated, TetraMito has only 66% prediction accuracy on the outer membrane class with a similar MCC value to our method. The only drawback of our method is the performance on matrix. The prediction accuracy is slightly lower than existing methods. However, the MCC on matrix location is still higher than most of the existing methods. Therefore, it is fair to say that our method is comparable to all state-of-the-art methods in predicting protein submitochondrial locations.

To further validate the performance of our method, we carried out an independent dataset test. For both SML3-983 and SML3-317 datasets, 80% sequences were randomly selected as the training dataset. The predictor was trained with these 80% sequences. The prediction performance was estimated using the remaining 20% sequences. These procedures were repeated 20 times for every dataset. The average prediction performance and the standard deviation of the accuracy were shown in Table 5. The independent dataset test performance is similar to the jackknife test performance. These results proved that the performance of our method was not overestimated.

### 3.2. Advantages of PSPCP

 In the method section, we have already described how to generate the PSPCP features. We will now discuss why we use (4) to define a replacement of physicochemical properties in the PseAAC.

 The protein functions, including its subcellular locations, are largely determined by the physicochemical properties of the residues on the sequence. However, not all residues contribute to the protein functions equally. Some of the residues are important, while others are not. In the evolutionary process, the important residues tend to be conserved, or at least can only vary to limited types that possess similar physicochemical properties. But the unimportant residues would not be conserved. Thus, we can assume that all unimportant residues would have similar replacement propensity patterns in the evolutionary history. Although it is difficult to figure out which residue is important and which is not, based on our assumption, the average physicochemical properties in the evolution history would be similar for all unimportant residues. Thus, if we compute the average physicochemical properties in the evolution history, the important residues would possess physicochemical properties that are much more different to those unimportant ones. This is why we use PSPCP, which is the average physicochemical properties of all residues in the evolution history, to replace the conventional physicochemical properties in the PseAAC.

Developing novel methods for predicting protein submitochondrial locations is not only a race of prediction performance. There are many different quality terms other than prediction accuracy that can be used to describe how good a prediction method is. There are two major advantages of our method, the simpleness and the potential to improve all existing PseAAC-based methods. 

The feature vectors in all state-of-the-art methods usually have several hundreds to over a thousand dimensions, which is a number much larger than the number of the samples in the benchmarking dataset. In the general concept of machine learning, a feature vector with lower dimensions is usually preferred when a similar performance can be achieved when other conditions are the same. Our method uses only about 100 dimensions feature vectors, which is lower in dimension than all existing methods except SubIdent.

Our method also has the potential to improve all existing PseAAC-based methods. Actually, the current method only replaces the physicochemical properties in the SubMito method with the PSPCP, which is derived from the same physicochemical properties in SubMito and the PSSM information. This simple replacement resulted in 8% performance improvement, which proved that the PSSM information is very useful in classifying protein sequences. Our method also gives a simple and effective way on how to integrate the PSSM information into all existing PseAAC-based methods. PsePSSM, which only extracts the information from PSSM, has achieved great success. Therefore, it can be anticipated that our method, which integrates PSSM within the PseAAC, could start a new way to utilize PSSM information more efficiently.

As pointed out by TetraMito, the GO-based methods usually achieve better performances, like Fan and Li's work. There is no doubt that GO-based methods are very useful in computationally determining protein subcellular locations. In the view of a user, today's GO-based methods require the same input as the sequence-based *ab initio* methods and provide a better result, which is very promising in practical studies. However, this cannot conceal the following fact. When a protein sequence was given to predict its locations, the performance of GO-based methods relies on whether similar sequences of the given sequence can be found in the UniProtKB database. Therefore, almost every existing GO-based method tried to incorporate some sequence-based information as its complement. Our method provides a perfect complement to the GO-based methods, as all GO-based methods, which used to incorporate PseAAC as the complement, can now be upgraded to use PSPCP within PseAAC. Actually, these methods can work side by side to help each other in a practical study. 

### 3.3. Software Availability

We have developed an online service called SubMito-PSPCP. This service can be accessed using the following URL: http://www.pufengdu.org/srv/bioinfo/submito-pspcp/. The datasets SML3-983 and SML3-317 can both be downloaded from the “download” page of this service. 

## 4. Conclusions

We developed a computational method that can predict the protein submitochondrial locations. We proposed the positional specific physicochemical properties concept and used this concept along with the pseudoamino acid compositions to generate protein descriptors. With only about 100 dimensions of the descriptors, we achieved comparable prediction performance to those methods using over a thousand descriptors. We hope this method can be an alternative choice in predicting protein submitochondrial locations.

## Figures and Tables

**Table 1:** Summary of the dataset.

Submitochondrial locations	Number of proteins	
	SML3-317	SML3-983
Inner membrane	131	661
Outer membrane	41	145
Matrix	145	177

Total	317	983

**Table 2:** Physicochemical properties used in this method.

AAIndex ID	Property description
BULH740101	Transfer free energy to surface
EISD840101	Consensus normalized hydrophobicity
HOPT810101	Hydrophilicity value
RADA880108	Mean polarity
ZIMJ680104	Isoelectric point
MCMT640101	Refractivity
BHAR880101	Average flexibility indices
CHOC750101	Average volume of buried residue
COSI940101	Electron-ion interaction potential values

**Table 3:** Prediction performance on SML3-983 dataset.

Submitochondrial location	ACC	MCC
Inner membrane	95.46%	0.77
Outer membrane	77.93%	0.83
Matrix	74.01%	0.73
Overall	89.01%	

**Table 4:** Performance comparison on SML3-317 dataset.

Methods	Inner membrane		Matrix		Outer membrane		Overall
	ACC	MCC	ACC	MCC	ACC	MCC	
SubMito [21]	85.50%	0.79	94.50%	0.77	51.20%	0.64	85.20%
GPLoc [22]	83.20%	0.80	97.20%	0.85	78.10%	0.77	89.00%
SubIdent [23]	91.60%	0.86	97.30%	0.79	82.90%	0.88	93.10%
Predict_SubMito [26]	91.80%	0.79	96.40%	0.79	66.10%	0.63	89.70%
MitoLoc [25]	97.70%	0.94	99.00%	0.93	68.30%	0.81	94.70%
Fan and Li [24]	94.70%	0.91	99.30%	0.96	80.50%	0.84	94.90%
TetraMito [17]	100.00%	0.90	96.60%	0.95	65.90%	0.79	94.00%
This work	98.60%	0.92	93.90%	0.89	70.70%	0.79	93.10%

**Table 5:** Independent dataset test of the current method.

Dataset	Average ACC	Standard deviation of ACC
SML3-317	90.24%	3.27%
SML3-983	87.17%	1.81%

The values in this table are obtained by 20 times 20% independent dataset test.
